# Self-Assembly of Temperature Sensitive Unilamellar Vesicles by a Blend of Block Copolymers in Aqueous Solution

**DOI:** 10.3390/polym11010063

**Published:** 2019-01-04

**Authors:** Jong Dae Jang, Changwoo Do, Joona Bang, Young Soo Han, Tae-Hwan Kim

**Affiliations:** 1 Neutron Science Research Center, Korea Atomic Energy Research Institute, 1045 Daedeok-daero, Yuseong-gu, Daejeon 34057, Korea; jdjang@kaeri.re.kr (J.D.J.); yshan@kaeri.re.kr (Y.S.H.); 2 Department of Chemical and Biological Engineering, Korea University, 145 Anam-ro, Sungbuk-gu, Seoul 02841, Korea; joona@korea.ac.kr; 3 Biology and Soft Matter Division, Neutron Sciences Directorate, Oak Ridge National Laboratory, Oak Ridge, TN 37831, USA; doc1@ornl.gov; 4 Department of Quantum System Engineering, Chonbuk National University, 567 Baekjei-daero, Deokjin-gu, Jeonju 54896, Korea

**Keywords:** block copolymer, micelle, biocompatibility, vesicle, polymersome

## Abstract

A self-assembled unilamellar vesicle, which can be used as a drug delivery system, was easily and simply fabricated using a blended system of Pluronic block copolymers. Controlling the hydrophilic mass fraction of block copolymers (by blending the block copolymer with a different hydrophilic mass fraction) and temperature (i.e., the hydrophobic interaction is controlled), a vesicular structure was formed. Small angle neutron scattering measurements showed that the vesicular structure had diameters of empty cores from 13.6 nm to 79.6 nm, and thicknesses of the bilayers from 2.2 nm to 8.7 nm when the hydrophobic interaction was changed. Therefore, considering that the temperature of the vesicle formation is controllable by the concentration of the blended block copolymers, it is possible for them to be applied in a wide range of potential applications, for example, as nanoreactors and nanovehicles.

## 1. Introduction

Since the 1990s, many studies have been conducted to investigate methods of manufacturing drug carriers using various types of nanomaterials. In order to achieve a drug delivery system with highly efficient and biodegradable properties suitable for the human body, it is very important to control the size of a drug delivery system [1,2]. Remarkable nanomaterials, containing surfactants, lipids, polymers, and inorganic materials, have been studied for application in drug delivery systems [3,4,5,6]. In particular, polymer-based vesicles (polymersome) have excellent properties for drug delivery, such as good entrapping efficiency, long-term stability, and easy handling.

Pluronic block copolymers (PEO_m_–PPO_n_–PEO_m_) with amphiphilicity are biocompatible materials, which are induced by the PEGylation effect [7,8,9]. Pluronic block copolymers also undergo different phase transitions depending on the temperature and are easily controlled, which can provide many self-assembled structures that are applicable for drug delivery. Considering that it is necessary for a drug transporter to have temperature sensitivity in the body, Pluronic block copolymers are able to be used as a human drug delivery system. However, it is very difficult to spontaneously form unilamellar vesicle structures using only Pluronic block copolymers [10,11,12,13,14].

Pluronic F127, one of the Pluronic triblock copolymers, can be used as a biocompatible material in therapeutic and pharmaceutical areas [15,16,17] and has been approved for use with food and drugs by Food and Drug Administrator (FDA) [1,18]. Pluronic F127 (PEO:PPO = 7:3) exists as a random coil below the critical micelle temperature (CMT) in an aqueous solution [19]. Since the CMT of Pluronic block copolymers can be controlled by the mass fraction of the Poly(ethylene Oxide) (PEO) block and the Poly(Propylene Oxide) (PPO) block, the specific temperature range for forming micellar structures is easily controlled by changing their mass fractions, which may provide a functional drug delivery vehicle with temperature sensitivity. Furthermore, upon controlling the mass fraction of the PEO block and the PPO block, we can expect different phase behaviors of block copolymers, providing various types of structures for drug delivery systems. In this study, to control the mass fraction of the hydrophilic part of Pluronic F127, Pluronic PE6200 (PEO:PPO = 2:8) was added to the F127 solution, which is helpful to induce the phase transition of the block copolymers with temperature sensitivity [20].

For analysis of the complex F127-PE6200, a series of small angle neutron scattering (SANS) measurements were performed, and then the intraparticle interference (which is the structural information of micelles) was obtained from the neutron intensities. The intensities revealed by SANS measurements were analyzed by core-shell model fitting. As a result, it was determined that the micelle phases of the F127-PE6200 block copolymer complex formed spheres, cylinders, and vesicles based on the concentration of PE6200 and temperature. Even though there have been a few studies regarding controlling the phase behavior of block copolymers, which arise from the changes in the surrounding conditions or different kinds of additives [21,22,23,24], in this study, the phase transition of the micelle was induced using block copolymers with identical types of blocks but different hydrophilic mass fractions. Mixing similar types of block copolymers helps to alleviate the heterogeneity of the material when they form a micelle, improving the affinity between each molecule. Therefore, the method is expected to contribute to an increase in biocompatibility for block copolymers, such as F127 conjugated medicine, which is currently being frequently used by the pharmaceutical industry for drug delivery applications. At the same time, vesicular micelles formed by phase transition are expected to be employed as carriers for catalysts in various fields and used as nanoreactors in nano- and biotechnologies [25,26,27,28]. Furthermore, this study can provide an easy method to fabricate the spontaneous unilamellar polymeric vesicles in an aqueous solution without complicated procedures.

## 2. Experimental Section

### 2.1. Materials

Pluronic block copolymers, F127 (PEO_99_PPO_65_PEO_99_, molecular weight = 12,600 g/mol) and PE6200 (PEO_10.5_PPO_30_PEO_10.5_, molecular weight = 2450 g/mol) were purchased from BASF (Ludwigshafen, German). The mass fractions of the hydrophilic moiety of the F127 and PE6200 block copolymers were 0.7 and 0.2, respectively. All chemicals were used without further purification. D_2_O (99.9 mol % deuterium enriched) was purchased from Cambridge Isotope Laboratory (Tewksbury, MA, USA).

### 2.2. Sample Preparation

The samples were prepared from a mixture of Pluronic F127 and PE6200 in aqueous solution (see Supporting Information Table S1). The concentration of Pluronic F127 was fixed at 0.25% by weight, and that of Pluronic PE6200 varied from 0% to 1.75% by weight (0, 0.25, 0.5, 0.75, 1.0, 1.25, 1.5, and 1.75 percent, respectively). To prepare a homogeneously-mixed solution of two kinds of block copolymers, the solutions were vigorously mixed by a vortex mixer for approximately 30 min. The sample mixture was labeled as F127_x_, where x is the concentration of PE6200.

### 2.3. Dynamic Light Scattering (DLS) Measurements

DLS measurements were carried out using a Zeta Plus particle size analyzer (λ = 659 nm, Brookhaven Instruments Corporation). DLS was applied to determine micellar hydrodynamic radius (*R*_h_) as a function of temperature. The DLS intensity autocorrelation function was measured and obtained using the cumulative method. The cumulative method was used to obtain an average decay rate and the second cumulant. The normalized values were measurements of the relative width of the distribution. The corresponding *R*_h_ was then calculated with the Stokes–Einstein equation [29].

### 2.4. Small Angle Neutron Scattering (SANS) Measurement

SANS measurements were carried out in a pinhole type SANS instrument at Oak Ridge National Laboratory (Oak Ridge, TN, USA). We used neutrons with a wavelength of λ = 6 Å. The *q*-range (where *q* = (4π/λ) sin (θ/2) is the magnitude of the scattering vector and θ is the scattering angle) was from 0.0027 Å^−1^ to 0.32 Å^−1^, sample to detector distance (SDD) = 20 m and collimation length = 20 m. In order to cover the full *q*-range of the 40 m SANS instrument, intensities measured in the high *q*-range and low *q*-range were overlapped for analysis. The corrected datasets were placed on an absolute scale using data reduction software provided by NIST (Gaithersburg, MD, USA). All SANS measurements were carried out in D_2_O.

### 2.5. Small Angle Neutron Scattering (SANS) Analysis

In order to analyze SANS intensity, it is necessary to determine the scattering length density (SLD). The SLD is defined as:

(1) $$\mathrm{SLD}=\frac{\sum_{i=1}^{n}b_{c_{i}}}{V_{m}}$$

where $b_{c_{i}}$ is the bound coherent scattering length of the ith n atoms in a molecule with a molecular volume ($V_{m}$).

In this study, the models applied for analyses were Gaussian coil, core-shell sphere, core-shell cylinder and spherical vesicle with core-shell. The average micelle orientations can be expressed with the form factor *P*(*q*). For Gaussian coils showing a random walk distribution, *v* is the excluded volume parameter from the Flory mean field theory of polymer solutions. The Gaussian coil model is defined below [30]:

$$P_{g}\left(q\right)=\left[\frac{1}{vU^{\frac{1}{2v}}}\gamma \left(\frac{1}{2v},U\right)-vU\frac{1}{vU^{\frac{1}{2v}}}\gamma \left(\frac{1}{v},U\right)\right]$$

$$U=\left(2v+1\right)\left(2v+2\right)\frac{q^{2}R_{g}^{2}}{6}$$

$$\left(a,x\right)=\int_{0}^{x}dt\;t^{a-1}\exp\left(-t\right)$$

In the core-shell model, micelles have a homogeneous core and a Gaussian chain on the surface, which can be reproduced to various form factors to form micelle, mostly-spherical, ellipsoidal, and cylindrical shapes. It was assumed that one unit forms the core of the micelles and the other the corona. The core was assumed to have a homogeneous SLD. In the model for the polymer chains in the corona, Gaussian chains are attached to a core of semi-flexible chains. The form factors were parameterized such that excess scattering of the corona and the core were consistent with the composition and density of the two separate block units of the copolymer. It was assumed that the amphiphilic block copolymer consisted of a block unit for which the solvent was poor and a block unit was good. The insoluble blocks formed a relatively compact core, whereas the soluble blocks formed the corona surrounding the core. The core-shell model is defined below [31]:

$$\begin{array}{c} P_{mic}\left(q\right)=N_{agg}^{2}\beta_{core}^{2}P_{core}\left(q\right)+N_{agg}\beta_{shell}^{2}P_{shell}\left(q\right)+2N_{agg}^{2}\beta_{core}\beta_{shell}S_{shell-core}\left(q\right)\\ +N_{agg}\left(N_{agg}-1\right)\beta_{shell}^{2}S_{shell-shell} \end{array}$$

where *P_core_*, *P_shell_*, etch for the core-shell sphere, and cylinder models are described in the Supporting Information.

## 3. Results and Discussion

**Figure 1:**
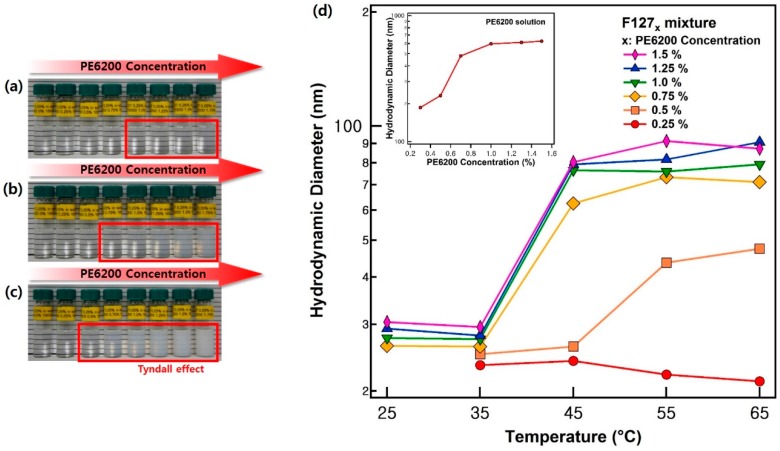
Visual inspection and dynamic light scattering (DLS) data. The figure on the left depicts the results of visual inspection. The temperatures in the figures are (**a**) 40 °C, (**b**) 45 °C, and (**c**) 50 °C. The concentration of PE6200 in each sample increased by 0.25% gradually from left to right. The concentration of PE6200 in the first sample is 0%. In the (**d**) DLS results, the graph on the right depicts the average size distribution of the micelles.

Since Pluronic F127 has a hydrophilic mass fraction of 70%, which can form spherical particles, its hydrophilic mass fraction should be decreased [20]. To induce the phase transition of Pluronic F127, it was blended with another Pluronic polymer, PE6200, which has the different mass fractions of the PEO block and the PPO block. The phase transition of the blended solution was visually confirmed. While the F127 (0.25%) solution showed transparent color at room temperatures, it changed to a bluish or white color, that is called the Tyndall effect, with an increasing amount of PE6200 and temperature (Figure 1a–c). It is natural that the blended solution indicate a bluish or white color when a large aggregate was formed in the solution. Therefore, this result strongly supports the idea that Pluronic F127_x_ forms large aggregates together with Pluronic PE6200 in solution [32,33].

In detail, when the temperature increased from 40 °C to 50 °C, Tyndall effects were observed in the F127_1.0_ and F127_0.75_ solutions and in the F127_0.5_ solution, respectively. Based on these results, the trend in the average size distribution was verified for each condition using DLS measurements (Figure 1d). The hydrodynamic diameters of F127_x_ are summarized in Figure 1d and Table S2 (Supporting Information). At 25 °C and 35 °C the particle sizes increased slightly with the amount of PE6200, and the average size distribution ranged from 20 nm to 30 nm. However, the average size distribution varied greatly with each concentration at temperatures above 45 °C. When the hydrodynamic diameter of F127x dramatically increased, the concentration of PE6200 was 1.5~0.75% and 0.5% for 45 °C and 55 °C, respectively, which is consistent with the results of the visual inspection. This means that the average size distribution of particles increased when the concentration of PE6200 and the temperature of the samples increased.

To understand the detailed phase behaviors of F127_x_ depending on temperature and the concentration of PE6200 polymer, a series of small angle neutron scattering measurements were carried out. The SLD of F127_x_ was determined by the scattering length of F127-PE6200 and the volume of F127-PE6200. The core and corona SLDs of F127x were 3.75 × 10^−7^ Å^−2^~3.59 × 10^−7^ Å^−2^ and 6.17 × 10^−7^ Å^−2^~5.75 × 10^−7^ Å^−2^, which are summarized in Table S3. The SLD of D_2_O was 6.38 × 10^−6^ Å^−2^. For the vesicular micelles with unilamellar vesicles, model fitting was carried out, simultaneously considering the hydrophobic and hydrophilic chains. The change in SLD due to changes in PE6200 concentration (from 0% to 1.25%) in the F127 solution was not more than 0.5%, while changes due to temperature were approximately 1%. As a result of model fitting of SANS data, changes in the SLDs with each concentration did not highly affect the results of reproducing SANS intensities. (Errors in micelle size due to the changes in SLD did not exceed ±5 Å). As a result of the SANS intensities, it was possible to predict that the micelle phase of the F127_x_ mixture changes as the temperature and x (concentration of PE6200) increase. It was predicted that the phase transition of the micelle went from a random coil at the beginning and gradually changed into a sphere, cylinder, and vesicle. Therefore, the SANS intensity of F127_0_ at 25 °C was reproduced using a Gaussian coil model, while the other intensities were reproduced using the core-shell model. The amphiphilic block copolymer consisted of the water insoluble core region and water-soluble corona region. Commonly, Pluronic block copolymers are well-known for which the random coil at room temperature is. The F127 random coil is mixed with PE6200, and the hydrophobicity increases as the temperature increase. As a result, the boundaries between the core and the corona gradually become clear, and the core region and the corona region are inherent [34]. The assumptions in this analysis were that all core-shell-shaped micelles were composed of homogeneous cores (PPO block) and Gaussian chains (PEO block) attached to the core [35,36,37]. Core radius and its polydispersity was described by applying the Schulz-Zimm distribution, which is suitable for describing polymer molecular weight distributions [38].

**Figure 2:**
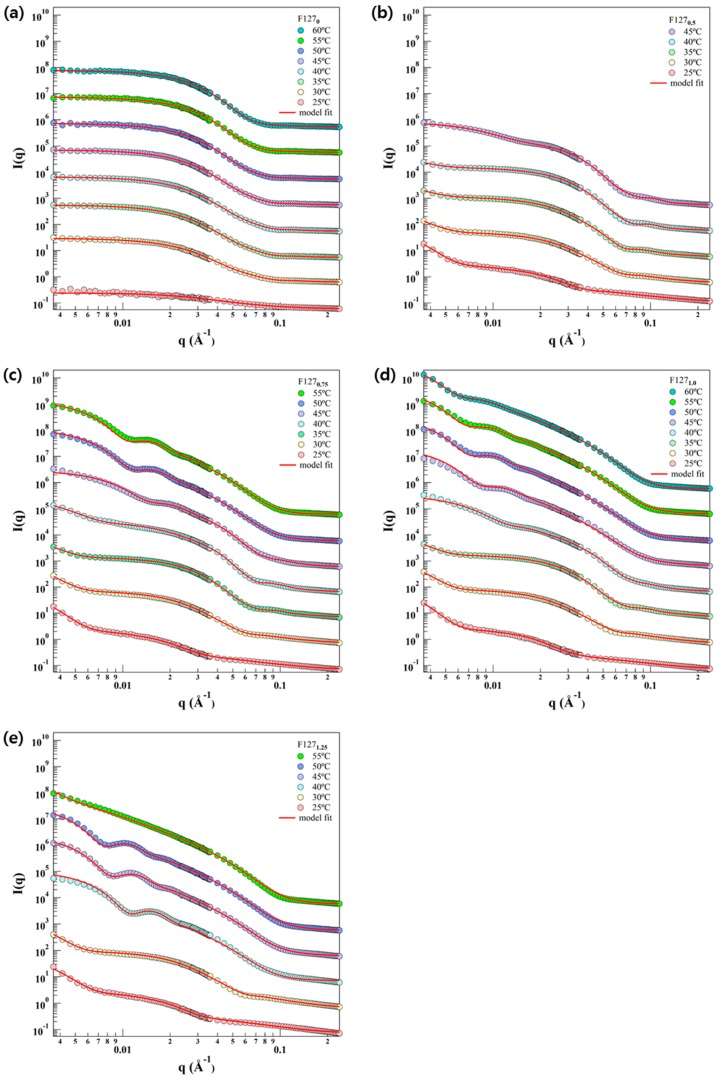
Small angle neutron scattering (SANS) intensities of the F127_x_ mixtures at various temperatures. SANS intensities of the F127_x_ mixture in water with increasing PE6200 concentrations at (**a**) F127_0_, (**b**) F127_0.5_, (**c**) F127_0.75_, (**d**) F127_1.0_, and (**e**) F127_1.25_ when the temperature was increased from 25 °C to 60 °C. SANS intensities have been vertically shifted for visual clarity.

**Figure 3:**
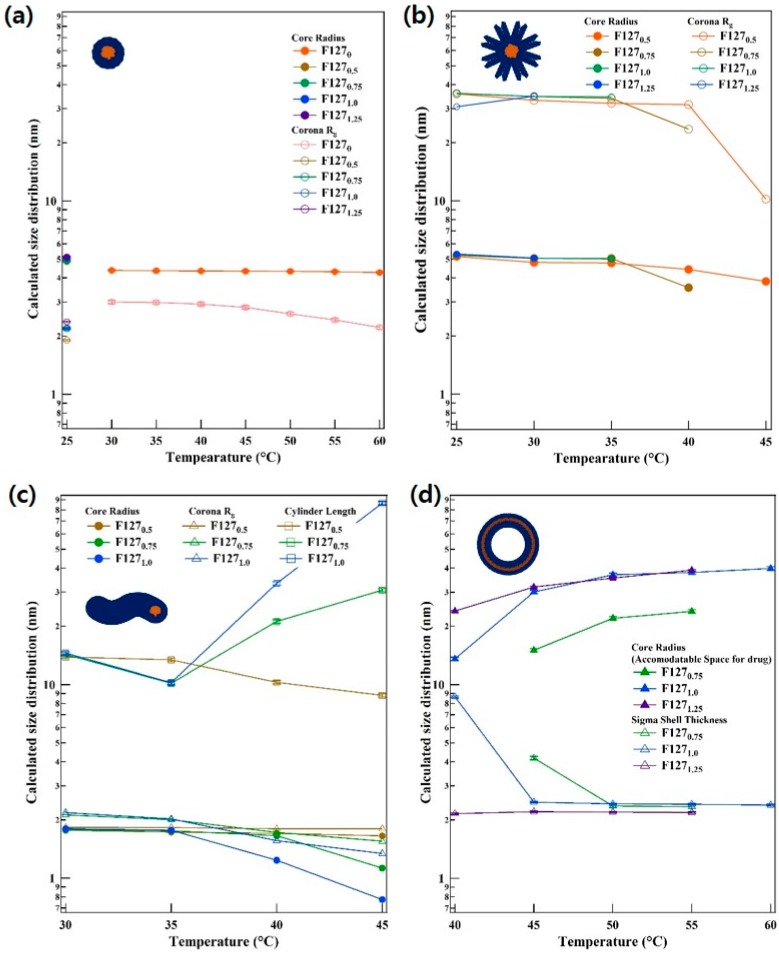
Information about nanostructure and intramicellar sizes for each temperature and concentration. (**a**) Spherical micelle, (**b**) spherical micelle with stretched corona (SC) chain, (**c**) cylindrical micelle, and (**d**) vesicular micelle.

**Figure 4:**
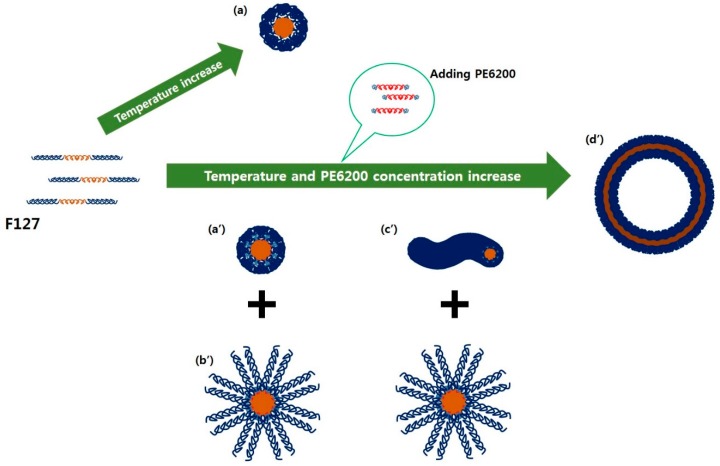
Phase transitions of F127_x_. (**a**) The F127_0_ is transformed into a spherical micelle. However, after adding PE6200, the other F127_x_ samples were transformed into (**a’**) spherical micelles, (**b’**) spherical micelles SC, (**c’**) cylindrical micelles, and (**d’**) vesicular micelle with temperature increase.

The SANS intensities were reproduced using the model (Figure 2). For F127_0_ at 25 °C, the SANS intensity was fairly low, which indicates that the F127 block copolymer exists in the form of a random coil in aqueous solution. The radius of gyration of the random coil polymer was obtained from the nonlinear squares model fit with the Gaussian coil model [30]. The size of coil for the Gaussian coil model for F127_0_ at 25 °C was approximately 5.22 nm. As the temperature increases, the SANS intensity of F127_0_ is increased as well, resulting in near *q*^−4^ behavior at the middle *q* region and a very flat scattering pattern at the low *q* region, which is typical for a spherical particle (Figure 2a), through the nonlinear squares model fit with the core-shell spherical form factor [31]. In addition, the intensities of F127_0.5_ at 30~45 °C, F127_0.75_ at 30~45 °C, F127_1.0_ at 30~40 °C, and F127_1.25_ at 30 °C showed *q*^−1^ behavior in low *q* region, which is typical for a cylinder form. The intensities of F127_0.75_ at 50~55 °C, F127_1.0_ at 45~60 °C, and F127_1.25_ at 40~55 °C showed *q*^−2^ behavior in the low *q* region, which is typical for a vesicular form [39]. However, in F127_0.5_ at 25~45 °C, F127_0.75_ at 25~45 °C, F127_1.0_ at 25~40 °C, and F127_1.25_ at 25~30 °C, a single model could not reproduce the SANS intensities. In these cases, the phases coexist before and after the transition and are regarded as being in the metastable region during the phase transition. Therefore, the intensities of the metastable region were reproduced by a sum of models, core-shell sphere and core-shell sphere with a different shell thickness, core-shell sphere and vesicle or core-shell sphere and cylinder. The micellar size information is depicted in Figure 3. Sizes were classified with respect to the form of micelles for each temperature, and the changes in them are aggregated in Figure 3a–d. Additionally, Figure 4 presents the transitions of the F127_x_ micelle form. It was revealed that the phase behavior of the F127_x_ micelle proceeds from sphere < cylinder < vesicle, which is a typical trend in polymeric micelle phase transitions [39,40].

Unusually, the F127_x_ micelle has two types of spherical forms (Figure 4a’) and (Figure 4b’). One is a spherical micelle like the one in Figure 4a’, while the other is a spherical micelle which has a stretched corona (SC) like (Figure 4b’). F127_0_ forms micelles with a spherical shape, where the corona and core chain were dehydrated, when the temperature increases. However, other F127_x_ samples with different concentrations showed that the corona of some micelles inside the solution tended to be stretched, even if the amount of PE6200 is slight. This phenomenon is considered to reflect a deepened partitioning between the core and corona due to increased hydrophobicity [41], indicating that the phase behavior is different for some micelles in the solution. At this time, although the status of the core itself remains the same with each shape, the partitioning of the core becomes stronger as hydrophobicity increases. As a result, the PEO block becomes dense with the temperature increase. On the other hand, some of the PE6200, which has high mass fraction of hydrophobic molecules, does not settle in the core region and is pushed toward the PEO chain, so that the corona appears stretched [24].

In addition, when there is a phase transition from spherical micelles to cylindrical micelles, SC changes into a vesicular micelle rather than changing into a cylinder. The data for F127_1.0_ at 35~40 °C shown in Figure 2 and Figure 3 and the Supporting Information (Table S4), indicate that SC gradually transformed into a vesicular micelle. This means that the phase transition of SC to a vesicle is faster than the transition to a cylindrical micelle. This is possible because SC is comparatively sensitive to hydrophobicity. In addition, the trend in basic phase transitions, except for SC, of which a small amount exists inside F127_x_ micelles, follows the phase transitions of basic polymeric micelles, as stated above. As a result, the distribution of micelle sizes determined by SANS intensity analysis almost match the DLS data depicted in Figure 3. Additionally, the most important result of this study is that micelle hydraulic diameter (*D*_H_), which appears when a vesicle exists as a single phase among F127_x_ micelles (F127_0.75_ at 50~55 °C, F127_1.0_ at 45~60 °C, and F127_1.25_ at 40~55 °C), has a size of approximately 50~85 nm. It has a small size and thus can be applied to the human body circulatory system. In addition, since they have space to accept materials with diameters of 40~80 nm inside their cores, they are expected to play important roles in the transfer of hydrophilic or neutral drugs, genes, or DNA.

## 4. Conclusions

In this study, a method to control the phases of polymeric micelles using an easy and simple method was introduced. Pluronic F127 triblock copolymer exists as a random coil and spherical micelle. It is very difficult to change it into other forms such as a cylindrical or vesicular micelle under a F127_0_ solution. However, the F127-PE6200 complex (F127_x_) system effectively induces a change in the form of its micellar shapes as sphere > cylinder > vesicle types. Even though it is very difficult to achieve a unilamellar vesicle, the F127_x_ complex system was able to obtain spontaneous unilamellar vesicles. Considering that the F127-based vesicle has a bilayer consisting of hydrophilic blocks (which are biocompatible) and an inner wall consisting of hydrophobic blocks (which prevent the loss of materials by hydrophobic cohesiveness), it is expected to be applied as a nano-scale carrier for drug delivery. In addition, the F127_x_ complex system presents a direction that can easily solve problems that are difficult to solve in F127_0_ and has shown very interesting results.

## Supplementary Materials

The following are available online at http://www.mdpi.com/2073-4360/11/1/63/s1.
