# Supplementary material for: Self-Assembly of Temperature Sensitive Unilamellar Vesicles by a Blend of Block Copolymers in Aqueous Solution
Source: Polymers (Basel). 2019 Jan 4;11(1):63. doi: 10.3390/polym11010063 (PMC6402003; doi:10.3390/polym11010063)
Supplement: Supplementary file 1 [file polymers-11-00063-s001.pdf]

Supporting Information

Self-Assembly of Temperature Sensitive Unilamellar Vesicles by a Blend of Block Copolymers in Aqueous Solution

Jong Dae Jang, Changwoo Do, Joona Bang, Young Soo Han, Tae-Hwan Kim

Table S1. The concentrations of the sample in solution (%)

	F127	PE6200
$F127_{0}$		0.00
$F127_{0.25}$		0.25
$F127_{0.5}$		0.50
$F127_{0.75}$	0.25	0.75
$F127_{1.0}$		1.00
$F127_{1.25}$		1.25
$F127_{1.5}$		1.50
$F127_{1.75}$		1.75

Table S2. The results of average size distribution by dynamic light scattering (DLS) (nm)

	25°C	35°C	45°C	55°C	65°C
$F127_{0.25}$	-	23.4	24.0	22.1	21.2
$F127_{0.5}$	-	25.0	26.2	43.6	47.5
$F127_{0.75}$	26.3	26.2	62.5	73.3	71.2
$F127_{1.0}$	27.6	27.4	76.5	75.9	79.3
$F127_{1.25}$	29.2	28.0	79.2	81.7	90.7
$F127_{1.5}$	30.4	29.5	80.3	91.3	87.2

Table S3. SLDs of core and corona of the micelle (Å^{-2})

	Core	Corona
F127$_0$	3.75 x 10^{-7}	6.17 x 10^{-7}
F127$_{0.5}$	3.62 x 10^{-7}	5.83 x 10^{-7}
F127$_{0.75}$	3.61 x 10^{-7}	5.79 x 10^{-7}
F127$_{1.0}$	3.60 x 10^{-7}	5.76 x 10^{-7}
F127$_{1.25}$	3.59 x 10^{-7}	5.75 x 10^{-7}

Table S4. Intramicellar information of F127$_x$ (nm)

Sample	T (°C)	Micelle Shape	Ratio	PDI$_{Rc}$	Core Radius	Corona Thickness	Cylinder Length	N_{agg}
F127$_{\mathbf{0}}$	30	Sphere	1	0.13	4.37	2.99		0.0007
	35	Sphere		0.16	4.36	2.99		0.0045
	40	Sphere		0.18	4.34	2.92		0.0060
	45	Sphere		0.19	4.33	2.82		0.0102
	50	Sphere		0.20	4.32	2.61		0.0137
	55	Sphere		0.020	4.30	2.43		0.0167
	60	Sphere		0.25	4.26	2.22		0.0197
F127$_{\mathbf{0.5}}$	25	Sphere	0.67	0.24	4.87	1.90		0.0003
	30	Cylinder		0.15	1.77	1.83	13.88	0.0027
	35	Cylinder		0.22	1.73	1.82	13.40	0.0086
	40	Cylinder		0.24	1.70	1.80	10.27	0.0241
	45	Cylinder		0.23	1.65	1.80	8.81	0.0313
	25	SC	0.33	0.04	5.15	35.80		0.0018
	30	SC		0.15	4.81	33.15		0.0438
	35	SC		0.12	4.77	31.98		0.0638
	40	SC		0.09	4.42	31.47		0.0890
	45	SC		0.20	3.83	10.22		0.1860
F127$_{\mathbf{0.75}}$	25	Sphere	0.75	0.38	4.93	2.18		0.0002
	30	Cylinder		0.19	1.78	2.12	14.27	0.0025
	35	Cylinder		0.16	1.76	2.00	10.16	0.0094
	40	Cylinder		0.31	1.66	1.72	21.16	0.0181
	45	Cylinder		0.54	1.13	1.55	30.73	0.0253
	25	SC	0.25	0.069	5.23	35.91		0.0032

	30	SC		0.14	5.04	34.67		0.0645
	35	SC		0.14	5.04	33.88		0.0855
	40	SC		0.42	3.56	23.49		0.2590
	45	Vesicle		0.21	15.02	4.18		0.0188
	50	Vesicle	1	0.25	21.98	2.36		0.0338
	55	Vesicle		0.24	23.86	2.34		0.1490
$F127_{1.0}$	25	Sphere	0.8	0.39	5.06	2.20		0.0002
	30	Cylinder		0.14	1.80	2.18	14.53	0.0021
	35	Cylinder		0.14	1.77	2.02	10.24	0.0077
	40	Cylinder		0.57	1.24	1.56	33.32	0.0198
	25	SC	0.2	0.31	5.28	36.02		0.0160
	30	SC		0.16	5.06	34.70		0.0806
	35	SC		0.16	5.01	34.53		0.0871
	40	Vesicle		0.20	13.56	8.67		0.0141
	45	Vesicle	1	0.27	30.02	2.47		0.0133
	50	Vesicle		0.24	36.85	2.42		0.0199
	55	Vesicle		0.26	37.94	2.41		0.0270
	60	Vesicle		0.30	39.77	2.39		0.0808
$F127_{1.25}$	25	Sphere	0.84	0.34	5.14	2.37		0.0002
	30	Cylinder		0.13	1.86	2.19	14.89	0.0019
	25	SC	0.16	0.16	5.31	30.70		0.0341
	30	SC		0.20	5.06	34.87		0.0807
	40	Vesicle	1	0.21	23.99	2.16		0.0301
	45	Vesicle		0.21	31.87	2.21		0.0216
	50	Vesicle		0.21	35.52	2.20		0.0294
	55	Vesicle		0.52	38.94	2.19		0.0407

(PDI_{Rc}: polydispersity of core radius, SC: stretched corona)

Spherical micelle model and vesicular micelle model (with core-shell); β_{core} of the vesicular micelle is 0.

$$P_{core}(q) = \left[\frac{3\,(\sin qR_{core} - qR_{core}\cos qR_{core})}{qR_{core}^3}\right]^2$$

$$P_{shell}(q) = \left[\frac{2\exp(-x) - 1 + x}{x^2}\right]^2 \qquad (x = q^2R_g^2)$$

$$\beta_{core} = V_{core}(\eta_{core} - \eta_{solv})$$

$$\beta_{shell} = V_{shell}(\eta_{shell} - \eta_{solv})$$

$$S_{shell-core}(q) = \Phi(qR_{core})\psi(qR_g)\frac{\sin(q[R_{core} + dR_g)}{q[R_{core} + dR_g]}\psi(qR_g) = \frac{1 - \exp(-x)}{x}$$

$$S_{shell-shell}(q) = \psi^2(qR_g)\left[\frac{\sin(q[R_{core} + dR_g)}{q[R_{core} + dR_g]}\right]^2$$

$$N_{agg} = (1 - x)\,\frac{(4\pi R_{core}^3)}{3V_{core}}$$

Cylindrical micelle model (with core-shell)

$$P_{core}(q) = \int_0^{\frac{\pi}{2}} \frac{4\sin^2(\frac{1}{2}q\,H\cos\alpha)}{q^2\,H^2\cos^2\alpha} \cdot \frac{4J_1^2\,(qR_{core}\sin\alpha)}{q^2R_{core}^2\sin^2\alpha}\sin\alpha\,d\alpha$$ (J_1: Bessel function of first order)

$$P_{shell}(q) = 2\,\frac{\exp(-x) - 1 + x}{x^2} \qquad (x = q^2R_g^2)$$

$$\beta_{core} = \frac{V(1 - x)}{N_{agg}}(\eta_{core} - \eta_{solv})$$

$$\beta_{shell} = V_{shell}(\eta_{shell} - \eta_{solv})$$

$$S_{shell-core}(q) = \psi(qR_g)\int_0^{\frac{\pi}{2}} \psi(q)\,\Theta(q, R_{core} + dR_g, H + 2d, \alpha)\sin\alpha\;\; d\alpha$$

$$\Theta(q) = [\frac{R}{R_{core} + H}\frac{2J_1(qR_{core}\sin\alpha)}{qR_{core}\sin\alpha}\cos\,(qH/2\;cos\;\alpha) + \frac{H}{R_{core} + H}J_0(qR_{core}\sin\alpha)\,\frac{\sin\frac{qH}{2}\cos\alpha}{\frac{qH}{2}\cos\alpha}$$

$$S_{shell-shell}(q) = \psi^2(qR_g) \int_0^{\pi/2} \Theta^2(q, R_{core} + dR_g, H + 2d, \alpha) \sin\alpha \; d\alpha$$

$$N_{agg} = n_{agg} S$$

$$S = 2\pi R_{core} H$$
